# Generation of microalga *Chlamydomonas reinhardtii* expressing shrimp antiviral dsRNA without supplementation of antibiotics

**DOI:** 10.1038/s41598-019-39539-x

**Published:** 2019-02-28

**Authors:** Patai Charoonnart, Nichakorn Worakajit, Julie A. Z. Zedler, Metha Meetam, Colin Robinson, Vanvimon Saksmerprome

**Affiliations:** 1 Center of Excellence for Shrimp Molecular Biology and Biotechnology, Mahidol University, Bangkok, 10400 Thailand; 2 National Center for Genetic Engineering and Biotechnology (BIOTEC) Thailand Science Park, Pathumthani, 12120 Thailand; 3 Department of Biology, Faculty of Science, Mahidol University, Bangkok, 10400 Thailand; 4 Centre for Molecular Processing, School of Biosciences, University of Kent, Canterbury, CT2 7NJ UK; 5 Copenhagen Plant Science Centre, Department of Plant and Environmental Sciences, University of Copenhagen, 1871 Frederiksberg C, Denmark

## Abstract

RNA interference (RNAi) is an effective way of combating shrimp viruses by using sequence-specific double-stranded (dsRNA) designed to knock down key viral genes. The aim of this study was to use microalgae expressing antiviral dsRNA as a sustainable feed supplement for shrimp offering viral protection. In this proof of concept, we engineered the chloroplast genome of the green microalga *Chlamydomonas reinhardtii* for the expression of a dsRNA cassette targeting a shrimp yellow head viral gene. We used a previously described chloroplast transformation approach that allows for the generation of stable, marker-free *C. reinhardtii* transformants without the supplementation of antibiotics. The generated dsRNA-expressing microalgal strain was then used in a shrimp feeding trial to evaluate the efficiency of the algal RNAi-based vaccine against the virus. Shrimps treated with dsRNA-expressed algal cells prior to YHV infection had 50% survival at 8 day-post infection (dpi), whereas 84.1% mortality was observed in control groups exposed to the YHV virus. RT-PCR using viral specific primers revealed a lower infection rate in dsRNA-expressing algae treated shrimp (55.6 ± 11.1%) compared to control groups (88.9 ± 11.1% and 100.0 ± 0.0%, respectively). Our results are promising for using microalgae as a novel, sustainable alternative as a nutritious, anti-viral protective feedstock in shrimp aquaculture.

## Introduction

The shrimp aquaculture industry remains threatened by significant losses due to several viral pathogens such as the yellow head virus (YHV) and the white spot syndrome virus (WSSV). RNA interference (RNAi) technology is a novel, highly effective technology for combating viral pathogens by using sequence-specific double-stranded (dsRNA) designed to knockdown the key viral genes. This method aims to harness the natural abilities of animals to combat pathogen infections. The application of this technology in small-scale tests suggests high efficiency of RNAi in controlling shrimp viruses that cause high mortality rates and slow growth syndrome^1,2^. For large-scale production, the utilization of a RNase III-deficient *Escherichia coli* strain expressing gene-specific dsRNA is efficient and inexpensive^1^. However, regular use of *E. coli* cells in shrimp feed has yet to be investigated for its long-term effect on the animals and the environment. Thus, finding alternative dsRNA production and delivery systems that can be safely used in shrimp farms is a key challenge for future application of this technology.

The eukaryotic green microalga *Chlamydomonas reinhardtii* has been investigated as a cell factory for the production of a wide range of biomolecules. The GRAS (Generally Regarded As Safe) status of this alga and the lack of any endotoxin production and infectious agents makes it particularly attractive^3^. Using this microalga as shrimp feedstock could, therefore, be an attractive alternative that would not raise concerns of health risk or environmental contamination^4^. Moreover, well-established genetic engineering tools for nuclear and chloroplast genome modification and gene expression are available for this microalga^5,6^.

The potential of dsRNA produced from the nuclear genome of *C. reinhardtii* to protect shrimps from YHV infection was previously shown^7^. Meanwhile, the chloroplast has become an attractive target for dsRNA production since it lacks any RNAi machinery for dsRNA processing allowing high-level accumulation of dsRNA within the organelle^8^. In addition, chloroplast transformation in *C. reinhardtii* occurrs via homologous recombination leading to integration of the gene of interest in a specific site whereas integration into the nuclear genome occurs randomly causing instability and issues with gene silencing^9^. More importantly, chloroplast transformation can be achieved by utilizing non-photosynthetic mutant strains, allowing for the generation of marker-free transformants that do not require antibiotics for selection^10^.

Economou *et al*. (2014) have established a simple and low-cost method for chloroplast transformation in *C. reinhardtii* by developing a non-photosynthetic (PSII^-^), cell wall-deficient *psbH*-knockout recipient strain (TN72)^11^. Transformation of this strain with a plasmid containing double homologous recombination loci and a functional *psbH* gene allows for insertion of the GOI using phototrophic growth as a selection pressure. The system has proven its practicality and reliable approach for producing several biochemical compounds such as a diterpene synthase and human growth hormone^12,13^. Thus, our aim was to exploit this system for shrimp antiviral dsRNA production in the *C. reinhardtii* chloroplast.

In this study we generated a stable, homoplasmic *C. reinhardtii* chloroplast transformant expressing a dsRNA specific to the YHV shrimp virus and demonstrated the efficiency of the algal transformant as feed supplement for shrimp disease control.

## Materials and Methods

### Strains and culture maintenance

The *Chlamydomonas reinhardtii* strain CC-5168 (cw15, ∆*psbH*, Spec^R^) a cell wall-deficient strain lacking a functional *psbH* gene, and thus PSII^−^, was used for chloroplast transformation (Chlamydomonas Resource Center, University of Minnesota, USA). The strain is also commonly referred to as “TN72”, which is how we will refer to it in this study. All algal strains were generally maintained by re-streaking on Tris Acetate Phosphate (TAP) pH 7.0 agar every 3 weeks and cultivated at 25 °C under low-intensity light. For all experiments in liquid culture, pre-cultures were prepared by inoculating liquid TAP medium from an agar plate and incubated for 4–5 days with continuously shaking at 100 rpm under low-intensity light.

### Construction of dsRNA-expression cassette and *C. reinhardtii* PYP strain generation by chloroplast transformation

**Figure 1:**
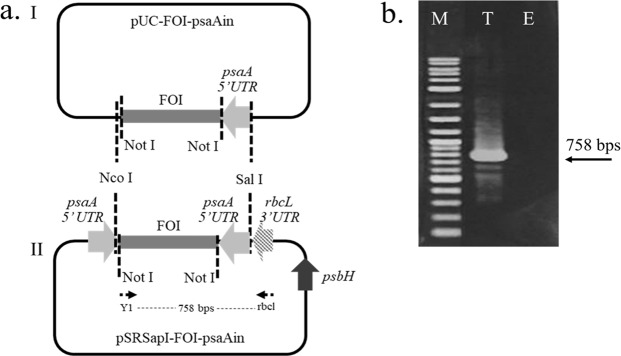
(**a**) Construction procedure of convergent promoter cassette expressing dsRNA. The cloning of fragment-of-interest (FOI) linked with an inverted *psaA* promoter (pUC-YHV_RdRp-psaAin) (I) into pSRSap I generating recombinant plasmid for chloroplast transformation (pSRSapI-FOI-psaAin) (II). (**b**) PCR analysis confirming insertion and direction of FOI-psaA in pSRSap I using primers Y1 and rbcl illustrated on the left whereas lane M is 2-log DNA marker, lane T represents pSRSapI-FOI-psaAin (pSR-PYP), and lane E represents pSRSapI.

A 374-bp fragment of the RNA-dependent RNA polymerase (RdRp) of the Yellow Head Virus (YHV) was used as target gene^1^. A double-stranded RNA expression cassette was designed using head-to-head promoters, so-called convergent promoters, for a sense-antisense expression approach (Fig. 1a). The construct was designed to obtain a fragment-of-interest (FOI) linked with an inverted *psaA* promoter at the downstream of the fragment. The designed fragment was custom-synthesized by GenScript (USA) (pUC-FOI-psaAin) (see Supplementary Material for gene sequence). The FOI-*psaA* sequence was then inserted into the plasmid pSRSapI^12^ suitable for *C. reinhardtii* TN72 chloroplast transformation. This plasmid was purchased from the *Chlamydomonas* Resource Center (University of Minnesota, USA). FOI-psaAin was cut and ligated with pSRSapI using the restriction sites NcoI and SphI (Fig. 1a). The correct plasmid assembly was confirmed by PCR and named pSRSapI-FOI-psaAin (pSR-PYP). The NotI restriction site was introduced for convenient replacement of the FOI with other viral targets.

Chloroplast transformation using glass beads was performed following a protocol described previously^11^. In brief, 10 mL of a pre-culture were inoculated into fresh 400 mL TAP medium and grown to an OD_750 nm_ between 1.0 and 1.2. The cells were then harvested and concentrated 100-fold. For each transformation, 300 µL of concentrated cells were transferred in a 5-ml tube containing approximately 300 mg acid washed and autoclaved glass beads (diameter: 400–625 µm). Five to ten micrograms of pSR-PYP plasmid were then added to each tube followed by vortexing at maximum speed for 15 s. Cells were immediately mixed with melted High salt medium (HSM) soft agar and directly poured on HSM agar plates. The plates were incubated in the dark overnight prior to illumination with 50 µmol photon m^−2^ s^−1^ for three to four weeks.

### Genotyping and homoplasmy analysis of *C. reinhardtii* transformants

**Figure 2:**
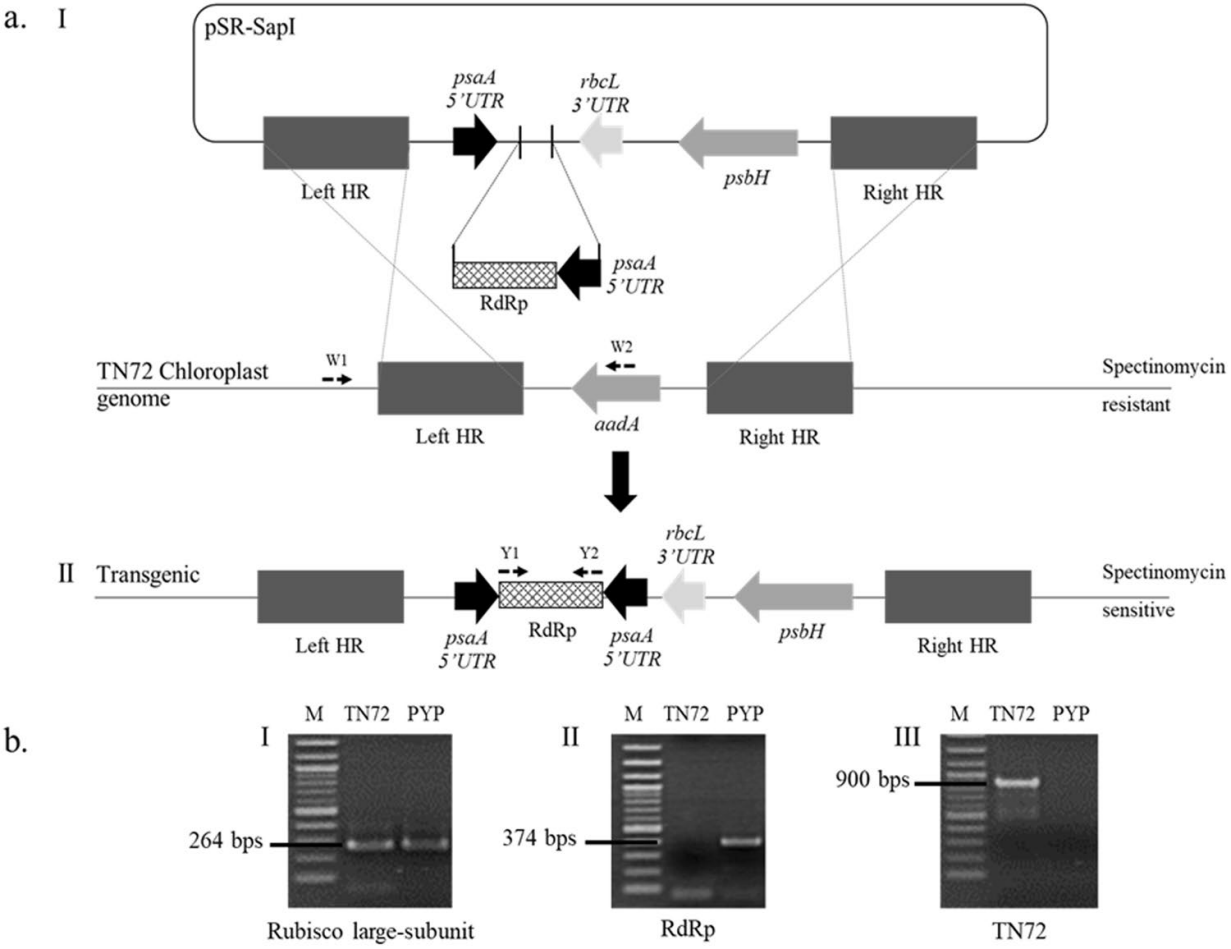
(**a**) Schematic diagram of chloroplast transformation. pSR-PYP containing *psbH* gene and convergent *psaA* promoters covering *RdRp* fragment was integrated into chloroplast genome (I) through double homologous recombination (left and right HR) replacing spectinomycin resistance gene (*aadA*) resulting spectinomycin sensitive and photosynthesis-restoration transgenic strain (II). (**b**) PCR analysis for selecting transformant containing dsRNA-expressed cassette; (I) qualification of chloroplast genome using Rubisco-large subunit (not shown in the diagram a) as internal control, (II) integration of dsRNA-expressed cassette using Y1 and Y2 primers, and (III) confirmation of homoplasmic status using W1 and W2, whereas lane M is 2-log DNA marker, lane TN72 and PYP represent amplicons resulting from genomic DNA extracted from TN72 and PYP, respectively.

The individual colonies obtained were picked and re-streaked on fresh HSM plates. After three passages, colonies were genotyped. Total genomic DNA was obtained by colony resuspension in Tris-EDTA (TE) buffer pH 8.0 and extraction of DNA using a CTAB based method^14^. As a control for successful chloroplast DNA extraction, the housekeeping gene *rbcL* (NCBI Accession No. BK00054.2) was amplified by Polymerase Chain Reaction (PCR) using the primers RBCL_F and RBCL_R (Table 1) (RBC Bioscience, Taiwan). Successful integration of the dsRNA-expression cassette was detected with FOI specific primers (Y1 and Y2). To confirm homoplasmy of the transformants, the TN72 chloroplast genome sequence using the primers TN72_F (W1) annealing upstream of the left flanking region and TN72_R (W2) annealing between the left and right flank (see Fig. 2a) was amplified. Homoplasmic strain was chosen and passaged on TAP agar for further use and named as PYP.

**Table 1:** List of primers used in this study.

Name in diagram	Primer names	Sequences	Function	References
Y1	YHV_F	5′ GCATGTCCTGTTCTC 3′	dsRNA-YHV (*RdRp*) specific primers (FOI)	^1^
Y2	YHV_Rin	5′ GAATTCTAGCCATGC 3′		
psaA	psaA_pro_F	5′ GCGTTGCTAATGGTGTAAAT 3′	dsRNA-expressing cassette checking	Own designed
rbcl	RbCL_ter_R	5′ CACATGCAGCAGCAAGTTCT 3′		
	RBCL_F	5′-GTCACCACCAGACATACGAAG-3′	Internal control for chloroplast DNA extraction	
	RBCL_R	5′ GGTCACTACTTAAACGCTAC 3′		
W1	TN72_F	5′ GTCATTGCGAAAATACTGGTGC 3′	Homoplasmic status checking (wildtype-derived primers)	^11^
W2	TN72_R	5′ CGGATGTAACTCAATCGGTAG 3′		
	GY2	5′ CATCTGTCCAGAAGGCGTCTATGA 3′	YHV detection (out of dsRNA specific region)	^7^
	GY3	5′ ACGCTCTGTGACAAGCATGAAGTT 3′		
	Actin_F	5′ CCTCGCTGGAGAAGTCCTAC 3′	Internal control for Shrimp RNA extraction	
	Actin_R	5′ TGGTCCAGACTCGTCGTACTC 3′

### Growth analysis

The growth of the generated *C. reinhardtii* strain expressing dsRNA-YHV (PYP) was analyzed in direct comparison to a control strain (TN72 transformed with the pSRSapI plasmid) previously generated^15^. Pre-cultures of both strains were prepared as described in Section 2.1 and 10 mL of each strain were re-inoculated into 400 mL TAP medium with continuous shaking at 100 rpm and 50 µmol photons m^−2^ s^−1^ illumination at 25 °C. Growth analysis was performed in triplicates by daily measurements of optical density at 750 nm until reaching stationary phase. Averages from biological triplicates were calculated, error bars show the standard deviation (n = 3).

### Specific dsRNA analysis and quantitation using RT-PCR and RT-qPCR

Total RNA of the PYP transformant was extracted at late-log phase from a 400-mL culture using TRIZOL (Ambion, USA). Double-stranded RNA was obtained by treating total RNA with DNase for 6 h at 37 °C to remove any genomic DNA, and RNase A for 1 h at 37 °C to remove any single-stranded RNA. The resulting dsRNA was confirmed by RT-PCR using the primers YHV_F and YHV_Rin (Table 1) (PCR Biosystem, UK). The presence of dsRNA was confirmed by digesting purified dsRNA with RNase III for 1 h followed by RT-PCR to observe dsRNA clearance.

To quantify dsRNA-YHV, 500 ng of treated total RNA was subjected to RT-qPCR (KAPA Biosystem, USA) using YHV_F and YHV_Rin primers. The sample was heated to 95 °C for 5 min, followed by immediate placement on ice for 1 min to unwind dsRNA prior to qRT-PCR^16^. A standard curve was prepared using ten-fold dilutions of known amount of dsRNA-YHV expressed from *E. coli* HT115^1^. Double-stranded RNA was calculated according to a formula obtained from the standard curve (R^2^ = 0.954).

### Shrimp feeding trial

*Penaeus vannamei* shrimp at post larval (PL) 30 were used for a feeding trial and a viral challenge test. The shrimp were acclimatized in 10 ppt of artificial seawater with aeration three days prior to the experiment. Shrimp were randomly divided into 4 groups (n = 25) with 3 replicates for each group cultivated in glass tank containing 2 L artificial seawater. Shrimp in groups 1 and 2 served as a negative (no algal treatment, no challenge) and a positive control (infection with YHV only), respectively. For Groups 3 and 4, 5 × 10^5^ cells per mL seawater of algal cells (*C. reinhardtii* strain SR and PYP, respectively) were added to the shrimp culture vessels every day. At day 3 of the experiment, shrimp in groups 2, 3 and 4 were fed with YHV-infected homogenized shrimp at 50% of their body weight. Daily survival of each group was observed for 8 days. The percentage of shrimp survival, at all sampling times, was calculated as the percentage of live shrimp from the initial shrimp group (n = 25). The data were analyzed using a One-Way ANOVA (SPSS ver.22, IBM).

After 8 days of cultivation, the surviving shrimp from each group were collected for evaluation of specific YHV inhibition by *C. reinhardtii* expressed dsRNA. Approximately 100 ng of total RNA was subjected to semi-quantitative RT-PCR using the primers GY2 and GY3 (Table 1), targeting the region outside of the dsRNA-YHV target. Actin-specific primers were used as an internal control.

## Results

### Generation of stable, marker-free *C. reinhardtii* strain expressing a specific dsRNA

The plasmid pSRSapI-FOI-psaAin with a dsRNA fragment specific to RdRp of YHV has been successfully generated and shown in Fig. 1b. This plasmid is compatible with chloroplast transformation of the *Chlamydomonas reinhardtii* strain TN72 using a glass bead method previously described^11^. The addition of a NotI restriction site in the plasmid allows for easy exchange of the FOI for other FOIs (Fig. 1a). Using the pSRSapI plasmid to transform the cell wall-deficient TN72 strain (*∆psbH*) allows for the selection of transformant colonies for phototrophic growth on HSM minimal medium by inserting a functional *psbH* gene together with the gene of interest while replacing the *aadA* antibiotic cassette present in TN72 (Fig. 2a).

After four weeks of incubation, one to two transformant colonies were obtained from transformation of TN72 with 5–10 µg pSR-PYP plasmid DNA. A 264-bp Rubisco large-subunit fragment was amplified with the extracted genomic DNA to assure consistent amount and quality of the extracted chloroplast DNA (Fig. 2b-I). Detection of integration of dsRNA-expressed cassette into the chloroplast genome was confirmed using FOI specific primers presenting 374-bp amplicon, while the wild-type strain did not yield any PCR product (Fig. 2b-II). Ensuring a state of homoplasmy was important for genetic stability of the transformant as *C. reinhardtii* contains approximately 80 copies of the chloroplast genome^17^. We confirmed homoplasmy of the generated transformant using PCR on the same genomic DNA amplifying both, the FOI and the TN72 genome copy (Fig. 2b II, III). The homoplasmic transformant strain we obtained was named PYP and subjected to further analysis. To assess the stability of the generated PYP strain given that no further selection pressure was applied once the strain was confirmed to be homoplasmic, we periodically checked for the presence of the dsRNA-expression cassette. The FOI in the PYP transformant still remained detectable after the 16^th^ passage (17 months after transformation).

### Double-stranded RNA has no significant impact on growth of PYP strain

**Figure 3:**
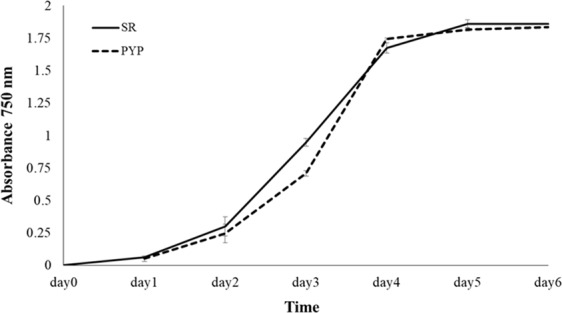
Growth rate according to absorbance at wavelength 750 nm of dsRNA-expressing *C. reinhardtii* strain (PYP,--) comparing to the control strain (SR, -). Bar represents standard deviation.

To investigate the impact on growth of dsRNA expression in the PYP strain, the growth of the strain was analyzed in comparison to a previously generated transformant that has the pSRSapI vector integrated in its chloroplast genome without a gene of interest (strain termed SR)^15^. This strain was chosen as a control since the TN72 strain used for transformation cannot grow phototrophically. Both SR and PYP strains showed an almost identical growth pattern in TAP medium (Fig. 3). Early-log phase was entered on day 2 and late-log phase was reached on day 4. No significant differences between the strains were observed.

### PYP strain produces dsRNA against YHV virus

**Figure 4:**
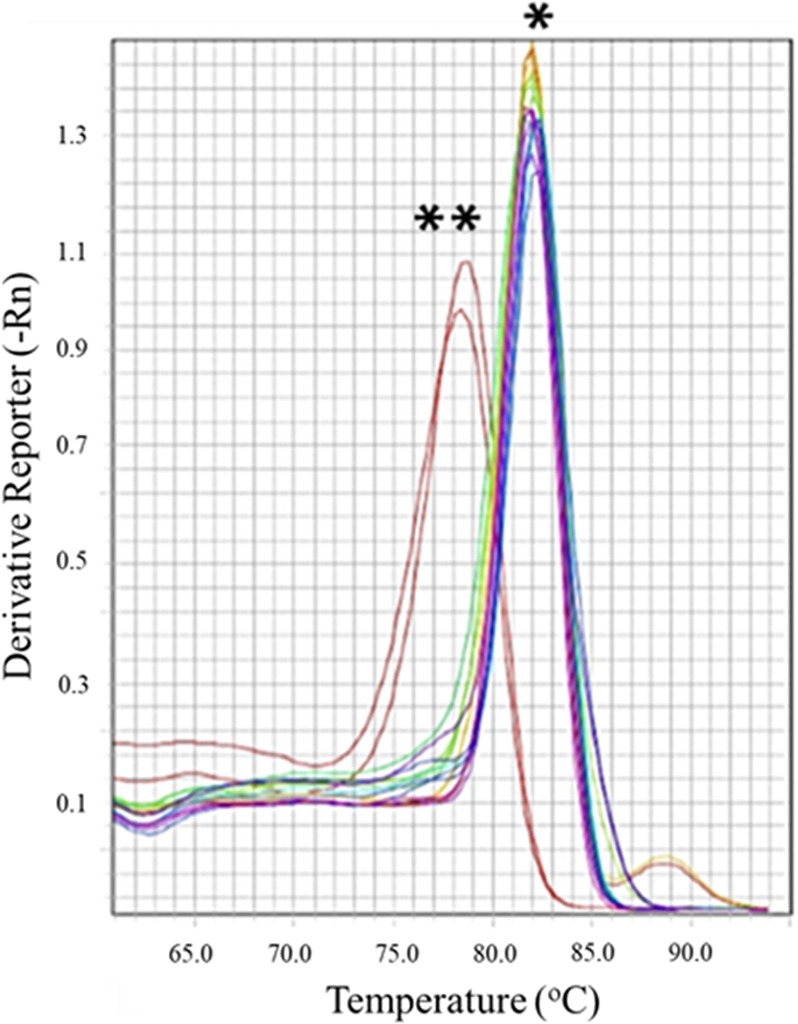
Confirmation of dsRNA by RT-qPCR by *represents melting temperature (T_m_) of dsRNA-YHV expressed from *E. coli* HT115 and dsRNA-YHV extracted from PYP strain while **represents negative reaction using ddH_2_O as a template.

To confirm the expression of the dsRNA cassette in the PYP strain, we performed RT-PCR with DNase and RNase treated RNA extracts from the PYP strain. A dsRNA-YHV fragment of 374-bp was amplified with RT-PCR and a negative control with the same RNA extract confirmed no DNA contamination (data not shown). Quantitation of microalgal dsRNA was performed using RT-qPCR. The melting temperature (Tm) of dsRNA from *C. reinhardtii* PYP was the same as the purified dsRNA used for the standard curve (81.9 ± 0.1 °C) (Fig. 4). One liter of *C. reinhardtii* PYP culture in late-log phase produced 16.0 ± 0.9 ng dsRNA.

### YHV-exposed shrimp survival rate increases using *C. reinhardtii* PYP as supplement

**Figure 5:**
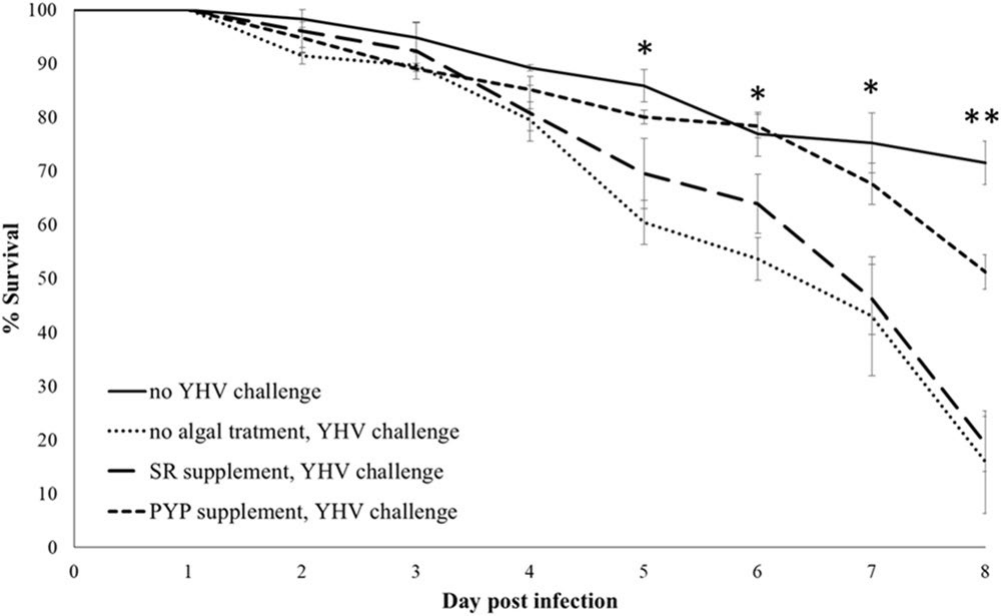
Post-larval shrimp survival percentage from feeding trial comparing between observed group (no YHV challenge) and normal feed (YHV challenge), SR supplement prior YHV challenge, and PYP supplement prior YHV oral challenge. Bar represents standard error. *and **Represent significant difference between experiment groups within observation day at confidential level p < 0.05 and p < 0.01, respectively.

To demonstrate the antiviral efficiency of the PYP transformant expressing the anti-YHV dsRNA, a feeding trial was performed by feeding shrimp with PYP algae prior to exposure to the virus. Figure 5 shows the survival percentage of shrimp fed with *C. reinhardtii* PYP relative to YHV-negative and positive controls. After challenging the shrimp by exposure to homogenized YHV-infected shrimp, the animals in the YHV-positive group died with 50% mortality at 6 days post-infection (d.p.i.) and reached 84.1 ± 16.7% mortality at 8 d.p.i. Survival in the YHV-negative group (no exposure to infected shrimp), on the other hand, remained significant at 70% on the last day of our experiment. Challenged shrimp fed with *C. reinhardtii* SR algal cells (no dsRNA-YHV expression) had a similar mortality rate to the YHV-positive group. Shrimp fed with the same amount of PYP algal cells, could however maintain a survival rate of up to 50% after 8 days. This was significantly different from the YHV-positive and the SR treatments at p < 0.01 confidence level (F value = 19.703, degrees of freedom = 3).

**Figure 6:**
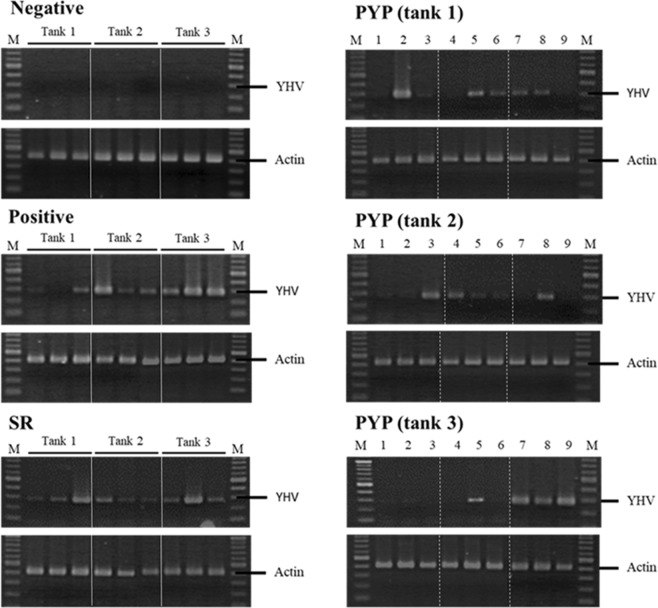
RT-PCR analysis detecting YHV infection of survival shrimps at 8 d.p.i. Actin specific primers were use as internal control. Lane M is 2-log DNA marker. Each lane represents infection level of individual PL shrimp. Solid lines separate 3 individual shrimps in each tank. Dash lines separate 3 individual shrimp in the same tank. Noted that there were more shrimps remaining in negative group while all remaining shrimp in the other groups was analyzed and presented in this figure.

RT-PCR of the YHV-positive group showed that 88.9 ± 11.1% of the 9 remaining shrimp were infected with YHV and of 100.0 ± 0.0% the remaining 9 shrimp in the SR-treated group were infected with YHV. In contrast, in the group treated with PYP, YHV infection levels were significantly lower (p < 0.05) with 55.6 ± 11.1% infection of the remaining 27 shrimps from all tank in this group (Fig. 6).

## Discussion

In this proof of concept study, we have successfully constructed an anti-YHV double-stranded RNA-expressing, marker-free *C. reinhardtii* chloroplast transformant. The goal of this study was to demonstrate the potential of using stable transgenic microalgae expressing dsRNA as a supplement for shrimp aquaculture. Due to the design of convenient FOI insertion and marker-free transformation system, this work can be used as an environmental-friendly model for producing dsRNA against other viruses.

Typically, previous studies used a hairpin DNA-structure approach^7,18^ for dsRNA expression, while we have decided to use convergent promoters for dsRNA expression in *C. reinhardtii*. There are several reasons for this choice: first, the recombination protein RECA found in plant chloroplasts (and also *C. reinhardtii*) might play a role in looping out the inverted repeats in the hairpin structure, thus leading to instability of the dsRNA-expression cassette^19,20^. Second, the use of convergent promoters allows to easily exchange the FOI to generate chloroplast transformation plasmids for the expression of other dsRNAs of interest. Regular checks for the dsRNA-expression cassette in the PYP transformant showed that very stable transformants were generated that do not need the application of further selection pressure. This, combined with the fact that no antibiotics are needed for stability, is an important attribute for large-scale applications of the transformant strains as aquaculture supplement.

The overall yield of dsRNA obtained in the PYP strain, was relatively low in comparison to what was obtained when expressing the dsRNA in *E.coli*^1^. However, the amount produced in the PYP microalgal strain, was still sufficient to offer viral protection to the shrimp in a daily whole-cell feeding dose of 0.17 pg per 25 PL shrimp. At the same time, the dsRNA expression level did not have a negative impact on growth of the *C. reinhardtii* strain (Fig. 3). We hypothesize that whole cell dsRNA delivery might contribute to protection of dsRNA from environmental degradation while potentially offering additional nutritional benefits conferred by algal consumption. Several studies have shown that only a few nanograms of a specific dsRNA can trigger target mRNA degradation resulting in viral copy number reduction. Alvarez-Sanchez *et al*. (2018) obtained a low amount of dsRNA from expression in the yeast *Yarrowia liolytica* with a maximum amount of 0.10% of total RNA (182 ng per liter culture)^21^. Intramuscular injection with 12 µg total RNA from this yeast could increase the survival of juvenile shrimp from White Spot Syndrome Disease (WSSD) up to 20% significantly different from control. Another study formulated feed with *Lactobacillus plantarum* expressing dsRNA thereby decreasing shrimp mortality from 100% in the control group to 70% in the dsRNA-treated group^22^.

In our feeding trial, the SR group (which was fed algal cells without dsRNA expression), showed a lower mortality rate between days 5 and 6, however, mortality then rose to similar levels as observed in the YHV-positive group. This might imply that *C. reinhardtii*, itself, confers nutrients or natural products, such as polyunsaturated fatty acids, to the shrimp aiding with combating the viral infection^23,24^.

To further improve the algal system presented in this proof-of-concept, using other promoters with previously reported high chloroplast transcription levels such as *psbA* or the 16S rRNA promoter may increase dsRNA yield and, consequently, improve shrimp viral protection^25^. In order to apply this algal supplement system in shrimp farms in the future, several aspects will have to be taken into consideration. One important point is the scalability of cell wall-deficient, genetically modified *C. reinhardtii* strains. It has previously been shown that transgenic strains, made with the same chloroplast transformation method as used in this study, can be scaled to 100 L photobioreactors^26^ providing first pilot-scale evidence that this microalgal dsRNA supplement could be implemented in larger scale reactors. Future studies will be needed to address public concerns on using living genetically modified organisms in aquaculture feed. For this, an algal cell inactivation process before addition to the shrimp feedstock should be considered.

To conclude, the dsRNA-expressing *C. reinhardtii* chloroplast transformants generated in this study have shown great potential as an effective way to fight viral diseases in shrimp aquaculture. Using whole algal cells as a shrimp supplement offers an easy and cost-effective oral delivery system. This system has great potential to be implemented in hatchery farms that produce PL shrimp. At this life stage, the shrimp are most susceptible to environmental changes and pathogens. Therefore, improving the overall resistance to pathogens of PL shrimp is, crucial for promoting sustainable farming.

## Supplementary information

Full-length gel and Custom-synthesized by GenScript (USA) (pUC-FOI-psaAin)
