# Supplementary material for: Generation of microalga Chlamydomonas reinhardtii expressing shrimp antiviral dsRNA without supplementation of antibiotics
Source: Sci Rep. 2019 Feb 28;9:3164. doi: 10.1038/s41598-019-39539-x (PMC6395707; doi:10.1038/s41598-019-39539-x)
Supplement: Supplementary file 1 — Full-length gel and Custom-synthesized by GenScript (USA) (pUC-FOI-psaAin) [file 41598_2019_39539_MOESM1_ESM.pdf]

Supplementary document 1

Generation of microalga *Chlamydomonas reinhardtii* expressing shrimp antiviral dsRNA without supplementation of antibiotics

Patai Charoonnart[1,2], Nichakorn Worakajit[3], Julie A.Z. Zedler[4], Metha Meetam[3], Colin Robinson[4], and Vanvimon Saksmerprome[1,2*]

*Corresponding author: Vanvimon Saksmerprome, Tel. +662-201-5870; Fax. +662-354-7344
Email: vsaksmer@gmail.com; vanvimon.sak@biotec.or.th

Full-length gels

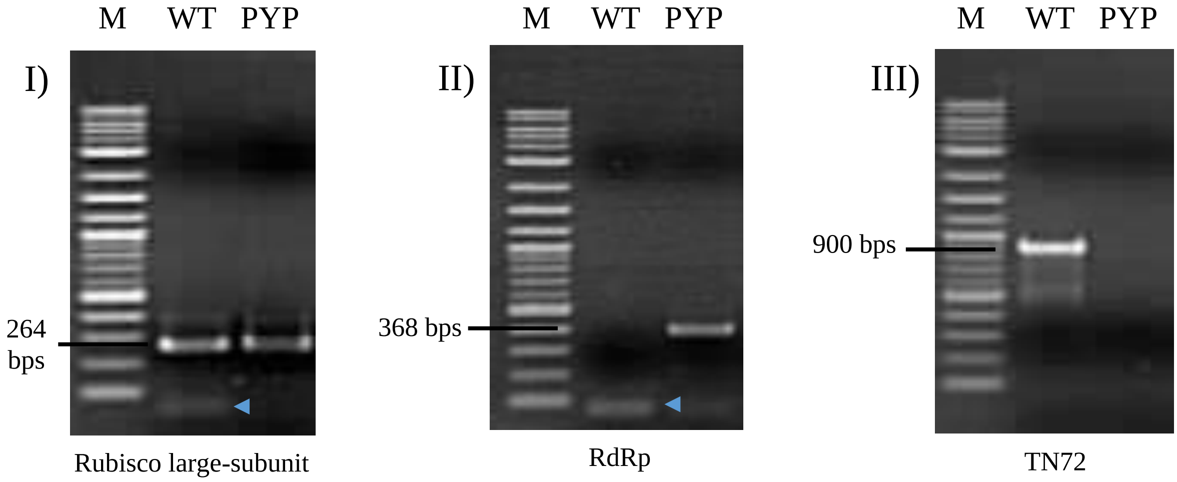

Full-length gels of Figure 2b in main article. Arrow heads represent primer dimer.

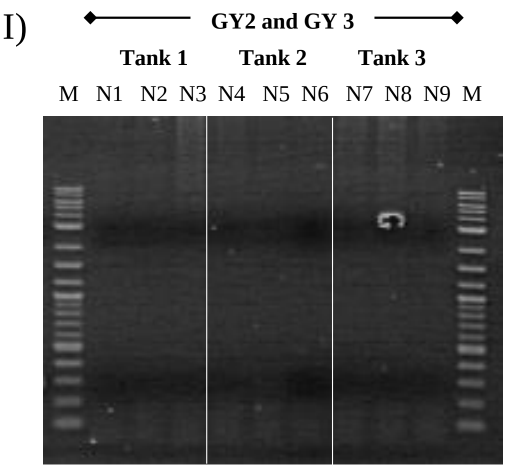
I)
GY2 and GY 3
Tank 1 Tank 2 Tank 3
M N1 N2 N3 N4 N5 N6 N7 N8 N9 M

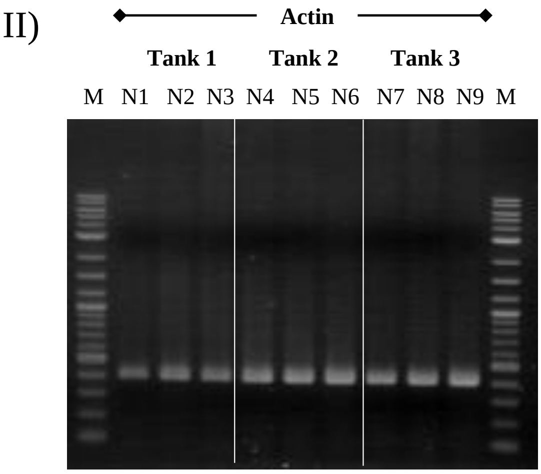
II)
Actin
Tank 1 Tank 2 Tank 3
M N1 N2 N3 N4 N5 N6 N7 N8 N9 M

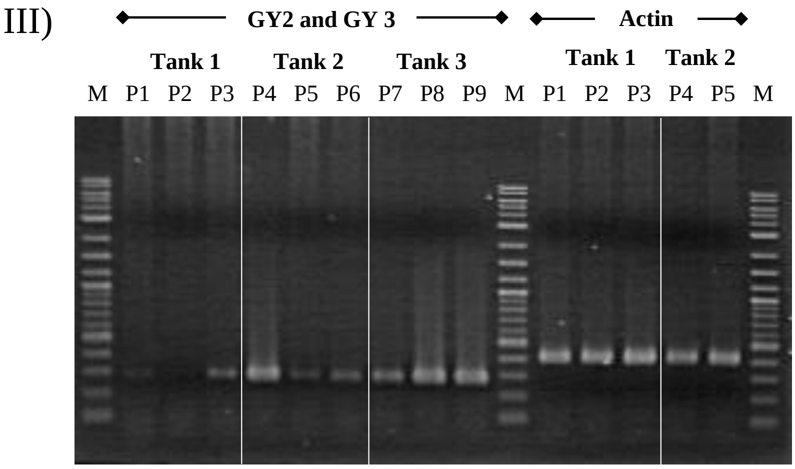
III)
GY2 and GY 3
Actin
Tank 1 Tank 2 Tank 3
Tank 1 Tank 2
M P1 P2 P3 P4 P5 P6 P7 P8 P9 M P1 P2 P3 P4 P5 M

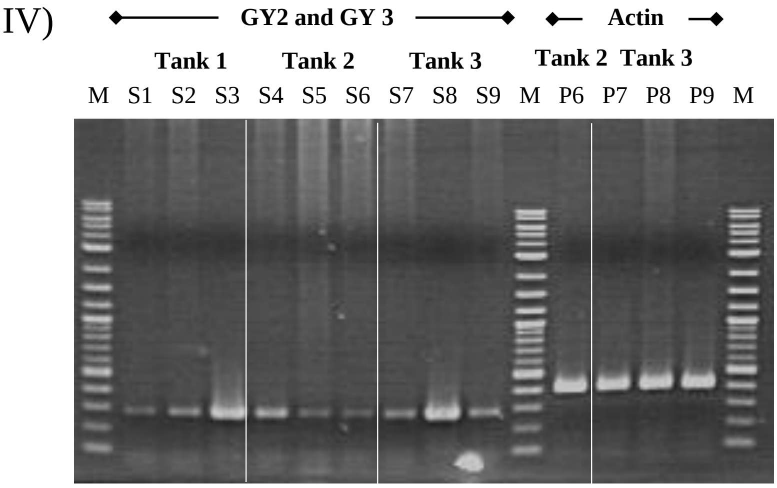
IV)
GY2 and GY 3
Actin
Tank 1 Tank 2 Tank 3
Tank 2 Tank 3
M S1 S2 S3 S4 S5 S6 S7 S8 S9 M P6 P7 P8 P9 M

V)

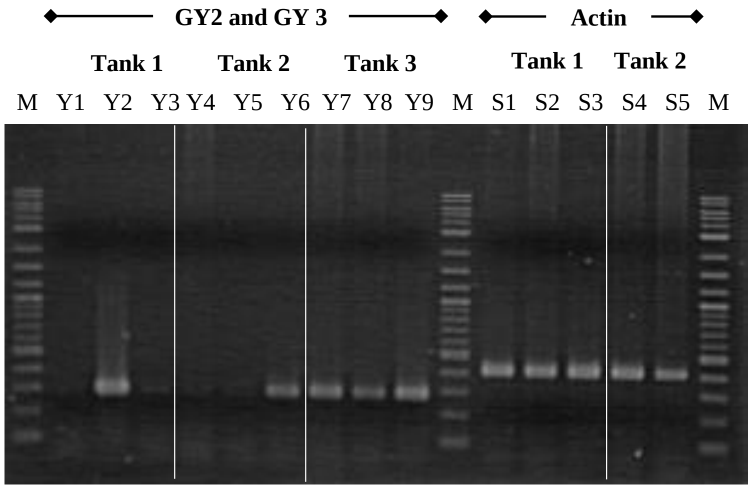
GY2 and GY 3
Actin
Tank 1 Tank 2 Tank 3
Tank 1 Tank 2
M Y1 Y2 Y3 Y4 Y5 Y6 Y7 Y8 Y9 M S1 S2 S3 S4 S5 M

VI)

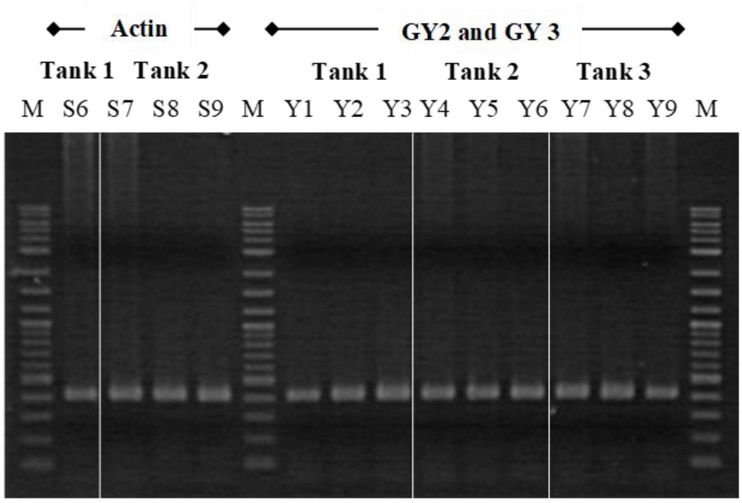
Actin
GY2 and GY 3
Tank 1 Tank 2
Tank 1 Tank 2 Tank 3
M S6 S7 S8 S9 M Y1 Y2 Y3 Y4 Y5 Y6 Y7 Y8 Y9 M

VII)

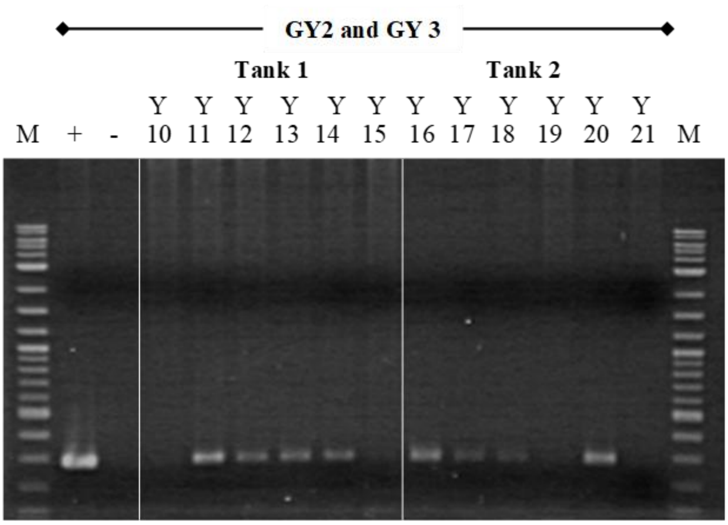
GY2 and GY 3
Tank 1 Tank 2
M + - Y 10 Y 11 Y 12 Y 13 Y 14 Y 15 Y 16 Y 17 Y 18 Y 19 Y 20 Y 21 M

VIII)

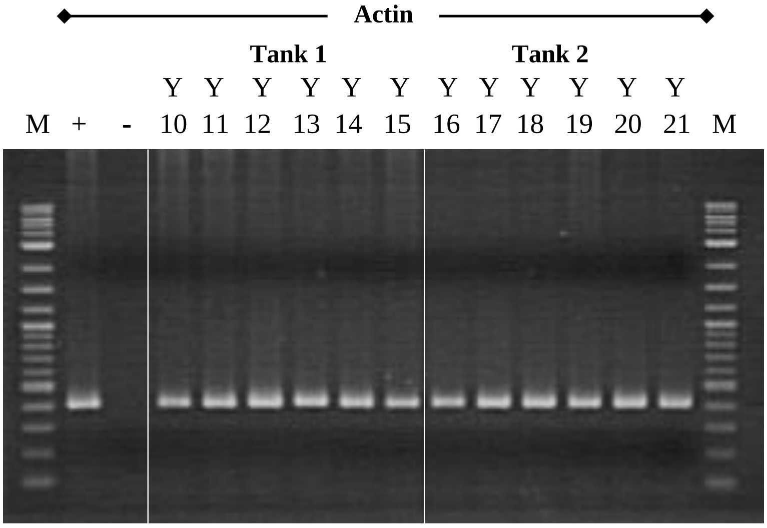

IX)

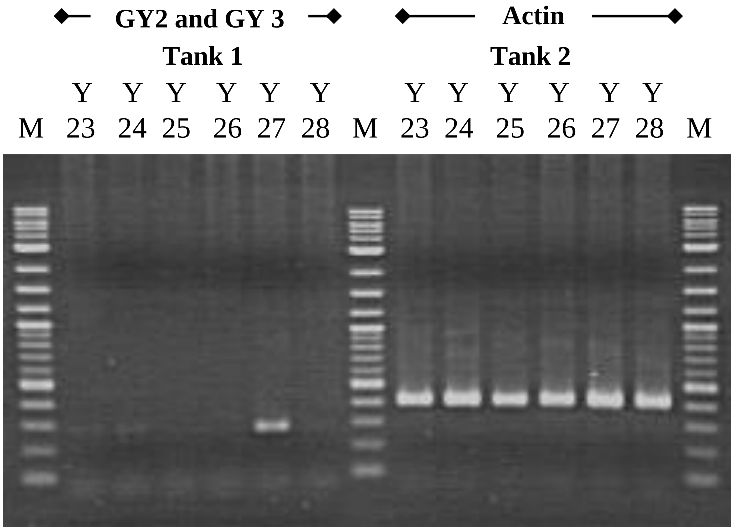

I-IX: Full-length gels from Figure 6. N1-N9: negative group; P1-P9: positive group; S1-S9: SR group; Y1-Y28: PYP group.

Supplementary document 2

Generation of microalga *Chlamydomonas reinhardtii* expressing shrimp antiviral dsRNA without supplementation of antibiotics

Patai Charoonnart[1,2], Nichakorn Worakajit[3], Julie A.Z. Zedler[4], Metha Meetam[3], Colin Robinson[4], and Vanvimon Saksmerprome[1,2*]

*Corresponding author: Vanvimon Saksmerprome, Tel. +662-201-5870; Fax. +662-354-7344
Email: vsaksmer@gmail.com; vanvimon.sak@biotec.or.th

Custom-synthesized by GenScript (USA) (pUC-FOI-psaAin)

5’-(Nco I)-(Not I)-
GCATGTCCTGTTCTCTCACTGAATTCCAGCTCTCTCTCTCTCACATCCTCTACCGT
TCTGAAGCACAGCGTACTCCTGACGACTTCCTCGACATAACACCTTACGAAGACA
CAAAGAAACCCGCATCCAAGAAATCTTCTGGCATCGGCATCACTAAACTCCCAC
AAGGCTATGTCCGATCACTCAGTGACTACACATCATTCATCAGATCACAAATCGA
ACACATCAAACACCACTTCTCTATCTGGCTATTTGAGGTGATTCCAAAGATCTCC
ATCCAACCTGTAGACAAAGCACTGCGTTCCATCTTCATCGGCCCAGCCTTCATGA
ACGATGTTTACCGCTGCTTCAACACCGCATGGCTAGAATTCACC-(Not I)-
GGATTTCTCCTTATAATAACAATTATTTAATTTAATAAATACATTATTTACACCAT
TAGCAACGCTTTTAATTATTATGTGTTCATAAAAAATGCATGGTTATTAAATTAG
ACAAATAGTTTTTTTACATCATATGTTATATTATACCATAAATATATTTTTTATT
GGTTTACAAATTATTTTTACTTATTTTAAAGATATTGTTTTATTTTTTACCATATGT
GTATTCGTAAAAGTATAACATTAAACAGTATTTACTTAAAAATGTTGAATTAAGA
AAGCTT-(Sph I)-3’

FOI (374 bps fragment of RNA-dependent RNA Polymerase gene of Yellow Head Virus)
Inverted sequence of psaA promoter and 5’UTR
